# Comparison of Children’s Oral Health Related Quality Of Life Pre- and Post Dental Treatment Under General Anesthesia Using F-ECOHIS Questionnaire

**Published:** 2013-11-05

**Authors:** Afsaneh Pakdaman, Seyed-Jalal Pourhashemi, Mahsa- Bagheri Ghalesalim, Sara Ghadimi, Yahya Baradaran-Nakhjavani

**Affiliations:** 1 Community Oral Health Department; 2 Pediatric Dentistry Department, School of Dentistry, Tehran University of Medical Sciences, Tehran, Iran

**Keywords:** Dental Caries, Anesthesia, Oral Health, Quality of Life

In developing countries, there is an increasing trend of chronic diseases such as tooth decay, obesity and diabetes especially in children[1]. Tooth decay in its severe form which is called nursing caries or early childhood caries (ECC) has impact on child growth and development. Children diagnosed with ECC weighed less than the matched control group due to the impact of pain and dysfunction on child’s eating and sleeping habit[2].

 Oral health related quality of life (OHRQoL) is measured by different tools[3,4]. In pre-school age children, Pahel developed ECOHIS questionnaire[5], which was validated in local languages[6]. Severe form of ECC (S-ECC) is defined as the presence of any sign of smooth-surface caries in children younger than 3 years of age or detecting 1 or more cavitated lesions in smooth surfaces of maxillary anterior teeth or more than 4 decayed, missing, or filled surfaces in 3-5-year old children[7].

 Parents of children under 5 years old attending three clinics providing complete dental treatment under general anesthesia (GA) consisting of Imam Khomeini Hospital, Mofid Children’s Hospital, and Shayamehr Clinics in Tehran, entered the study after ethical clearance and asked to fill in the validated Farsi version of ECOHIS questionnaire (n=81). Cases referred had severe caries, however were re-examined by two independent pediatric

dentists to confirm the need for treatment under GA. Those children having severe medical conditions and mental retardation were excluded. On the 1^st^ and 2^nd^ follow-up sessions 4 weeks and 3-months after dental rehabilitation the same family member who spent most of the time with the child was asked to fill in the same questionnaire. 

 The questionnaire contains 13 questions in two sections. Child section consists questions in four domains: (i) Child Symptom Domain (CSD) includes pain, (ii) Child Function Domain (CFD) includes child trouble in eating and drinking, pronouncing words and missing preschool or day care, (iii) Child Psychology Domain (CPD) includes trouble sleeping and being irritable, (iv) Child Self-image and Social Interaction Domain (CSID) includes avoiding smile or talk. Parent section was comprised (i) Family Distress Domain (FDD) and (ii) Family Function Domain (FFD)^[^^8^^]^. 

 Responses were from “never” to “very often” scored 0 to 4 and summed up in each domain for mean value calculation. Higher value in each domain demonstrates perceived negative impact of the oral health condition and compared on the baseline and follow up sessions. 

 In this study, significant improvement was observed in both child and parent sections. In the CFD domain the mean score 5.08±3.5 was significantly improved in the follow up sessions (Table 1). Abanto et al, have also reported high mean score of 4.15±3.92 in this domain among Brazilian children with S-ECC^[^^9^^]^. 

**Table 1:** Comparison of the Mean (Standard Deviation) ECOHIS score in each domain in the whole sample, pre and post treatment [minimum and maximum score of each domain]

**Domain**	**Pre- anesthetic**	**1-Month follow up**	**3-month follow up**
**CSD [0-4]**	1.8 (1.2) (n=79)	0.29* (0.71) (n=75)	0.061^‡^ (0.31) (n=49)
**CFD [0-16]**	5.08 (3.5) (n=71)	0.48* (0.95) (n=70)	0.02 (0.14) (n=48)
**CPD [0-8]**	3.2 (2.4) (n=79)	0.26* (0.95) (n=70)	Not reported
**CSID [0-8]**	0.97 (1.8) (n=76)	0.13*(0.68) (n=75)	Not reported
**FDD [0-8]**	5.5 (2.1) (n=77)	0.34* (1.08) (n=76)	Not reported
**Child [0-36]**	10.88 (7.6) (n=67)	0.98* (2.3) (n=65)	0.4 (0.2) (n=46)
**Parent [0-16] **	9.5 (3.5) (n=74)	2.4* (2.2) (n=73)	0.8 (1.32) (n=50)

* *P*<0.05; wilcoxon Signed Ranks Test

‡ *P*<0.05; Friedman Test

This implies caries in its severe form has impact on children’s usual daily function. There was also improvement in CSD domain after treatment. It was reported in the study of Acs et al, that improvement in painwas predominant followed by improved abilities to eat and sleep, reported by 86, 69, and 41% of parents, respectively^[^^10^^]^. Higher mean score of family distress domain, compared with child related domains was observed in this study in line with the study of Abanto et al, who reported the mean score of 3.43±2.59^[^^9^^]^. Filstrup et al, using the Michigan scale parents of children with ECC, evaluated their children’s oral health-related quality of life worse than that of children without ECC and they were able to realize that dental treatment of ECC improved children’s oral health-related quality of life^[^^11^^]^.

 This study showed significant improvement in oral-health related quality of life of children and especially their parents after dental rehabilitation. However, this should not be considered as a good reason to postpone prevention and promote treatment under general anesthesia in children.
